# Supplementary material for: Polyketide synthase gene clusters in the ericoid mycorrhizal fungus Oidiodendron maius: genome-wide prediction, expression profiling, and functional characterization of knockout mutants
Source: Front Plant Sci. 2026 Sep 16;17:1924430. doi: 10.3389/fpls.2026.1924430 (PMC13623903; doi:10.3389/fpls.2026.1924430)
Supplement: Supplementary file 1 [file SupplementaryFile1.zip › Supplementary Materials/Supplementary Folder 1.zip!/Oidma1_AssemblyScaffolds_Repeatmasked/knownclusterblast/cluster53/jgi.p_Oidma1_67262_mibig_hits.html]

| MIBiG Protein | Description | MIBiG Cluster | MiBiG Product | % ID | % Coverage | BLAST Score | E-value |
| --- | --- | --- | --- | --- | --- | --- | --- |
| ctg1\_orf003 |  | BGC0000334 | Cyclosporin\_biosynthetic\_gene\_cluster | 33 | 102.10748156 | 474 | 2.5e-133 |
| EFY94500.1 | non-ribosomal\_peptide\_synthetase | BGC0000337 | Destruxins\_biosynthetic\_gene\_cluster | 34 | 102.634351949 | 469 | 8.1e-132 |
| ABB90279.1 | non-ribosomal\_peptide\_synthetase | BGC0001057 | Zearalenone\_biosynthetic\_gene\_cluster | 33 | 101.58061117 | 453 | 7.8e-127 |
| EPE34341.1 | Acetyl-CoA\_synthetase-like\_protein | BGC0001035 | Pneumocandin\_biosynthetic\_gene\_cluster | 34 | 104.320337197 | 451 | 2.3e-126 |
| CAP93139.1 |  | BGC0000357 | Fungisporin\_biosynthetic\_gene\_cluster | 32 | 103.793466807 | 445 | 1.2e-124 |
| AFN69082.1 | nonribosomal\_peptide\_synthetase\_6 | BGC0001249 | Dimethylcoprogen\_biosynthetic\_gene\_cluster | 33 | 102.212855638 | 439 | 8.9e-123 |
| OAQ83772.1 | nonribosomal\_peptide\_synthase | BGC0001358 | Leucinostatins\_biosynthetic\_gene\_cluster | 33 | 105.584826133 | 439 | 1.2e-122 |
| CRG85572.1 | nonribosomal\_peptide\_synthase,\_putative | BGC0001402 | Cyclochlorotine\_biosynthetic\_gene\_cluster | 31 | 102.739726027 | 439 | 1.2e-122 |
| AFT91378.1 | EcdA | BGC0000338 | Echinocandin\_B\_biosynthetic\_gene\_cluster | 33 | 103.898840885 | 437 | 4.4e-122 |
| ABM91454.1 | lysergyl\_peptide\_synthetase\_LpsB | BGC0000348 | Ergovaline\_biosynthetic\_gene\_cluster | 32 | 104.214963119 | 436 | 9.9e-122 |
| ctg1\_orf000000 |  | BGC0000901 | Ferrichrome\_biosynthetic\_gene\_cluster | 33 | 102.950474183 | 435 | 1.7e-121 |
| BAN91483.1 | putative\_Aculeacin\_A\_nonribosomal\_peptide\_synthase | BGC0001220 | Aculeacin\_A\_biosynthetic\_gene\_cluster | 33 | 104.531085353 | 435 | 2.2e-121 |
| AMM63162.1 | AniA | BGC0001371 | Echinocandin\_B\_biosynthetic\_gene\_cluster | 32 | 102.950474183 | 426 | 6e-119 |
| AGC83576.1 | NRPS | BGC0000818 | Notoamide\_biosynthetic\_gene\_cluster | 32 | 103.477344573 | 422 | 1.1e-117 |
| ADM34138.1 | non-ribosomal\_peptide\_synthetase | BGC0001084 | Notoamide\_/\_stephacidin\_biosynthetic\_gene\_cluster | 32 | 103.582718651 | 417 | 3.6e-116 |
| CBF87069.1 | nonribosomal\_peptide\_synthase,\_putative\_(Eurofung) | BGC0001290 | Emericellamide\_A\_/\_emericellamide\_B\_biosynthetic\_gene\_cluster | 31 | 106.006322445 | 417 | 3.6e-116 |
| BAO73252.1 | non-ribosomal\_peptide\_synthetase | BGC0000456 | Verlamelin\_biosynthetic\_gene\_cluster | 30 | 103.582718651 | 416 | 8.1e-116 |
| EAL89049.1 | nonribosomal\_peptide\_synthase,\_putative | BGC0000355 | Fumiquinazolines\_biosynthetic\_gene\_cluster | 32 | 101.685985248 | 408 | 2.9e-113 |
| ADY16697.1 | TqaA | BGC0001142 | Tryptoquialanine\_biosynthetic\_gene\_cluster | 31 | 105.900948367 | 408 | 2.9e-113 |
| ACZ66258.1 | APS1 | BGC0000304 | Apicidin\_biosynthetic\_gene\_cluster | 31 | 103.688092729 | 400 | 4.6e-111 |
| AGQ43600.1 | HC-toxin\_synthetase | BGC0001166 | HC-toxin\_biosynthetic\_gene\_cluster | 31 | 104.531085353 | 387 | 4e-107 |
| AAF01762.1 | AM-toxin\_synthetase | BGC0001261 | AM-toxin\_biosynthetic\_gene\_cluster | 31 | 104.214963119 | 387 | 5.3e-107 |
| EAW16180.1 | nonribosomal\_peptide\_synthase,\_putative | BGC0000293 | Acetylaszonalenin\_biosynthetic\_gene\_cluster | 31 | 102.002107482 | 386 | 6.9e-107 |
| XP\_001227984.1 | hypothetical\_protein | BGC0001240 | Serinocyclin\_A\_/\_serinocyclin\_B\_biosynthetic\_gene\_cluster | 32 | 90.937829294 | 376 | 7.1e-104 |
| AAA33023.2 | HC-toxin\_synthetase | BGC0000370 | HC-toxin\_biosynthetic\_gene\_cluster | 30 | 106.111696523 | 363 | 1.1e-99 |
| BAH23995.1 | nonribosomal\_peptide\_synthetase | BGC0000356 | Fumitremorgin\_biosynthetic\_gene\_cluster | 29 | 103.898840885 | 353 | 8.4e-97 |
| CAI59267.1 | peptide\_synthetase\_3 | BGC0001263\_c2 | Ergometrine\_biosynthetic\_gene\_cluster | 29 | 105.163329821 | 347 | 3.5e-95 |
| CAP96445.1 |  | BGC0000420 | Roquefortine\_C\_/\_meleagrin\_biosynthetic\_gene\_cluster | 28 | 103.793466807 | 335 | 2.4e-91 |
| AGA37269.1 | NRPS | BGC0000819 | Paraherquamide\_biosynthetic\_gene\_cluster | 28 | 105.479452055 | 326 | 8.5e-89 |
| EAL89046.1 | nonribosomal\_peptide\_synthase,\_putative | BGC0000355 | Fumiquinazolines\_biosynthetic\_gene\_cluster | 30 | 104.425711275 | 326 | 8.5e-89 |
| ACI30655.1 | BEAS\_beauvericin\_nonribosomal\_cyclodepsipeptide\_synthetase | BGC0000313 | Beauvericin\_biosynthetic\_gene\_cluster | 32 | 69.7576396207 | 286 | 1.3e-76 |
| AGA37267.1 | NRPS | BGC0000816 | Malbrancheamide\_biosynthetic\_gene\_cluster | 35 | 56.5858798736 | 280 | 6.9e-75 |
| AFJ14794.1 | PlpE | BGC0000403 | Pelgipeptin\_biosynthetic\_gene\_cluster | 34 | 67.1232876712 | 262 | 2e-69 |
| ACZ55942.1 | non-ribosomal\_peptide\_synthetase | BGC0000302 | Anabaenopeptin\_biosynthetic\_gene\_cluster | 26 | 96.5226554268 | 262 | 2e-69 |
| AHJ31215.1 | Long-chain-fatty-acid--CoA\_ligase | BGC0000430 | Spumigin\_biosynthetic\_gene\_cluster | 26 | 102.423603793 | 261 | 4.4e-69 |
| CAE15637.1 |  | BGC0001128 | Luminmide\_biosynthetic\_gene\_cluster | 28 | 104.952581665 | 259 | 1.7e-68 |
| CAG29032.1 | nonribosomal\_peptide\_synthetase\_(modules\_3\_to\_6) | BGC0001023 | Myxochromide\_biosynthetic\_gene\_cluster | 28 | 103.898840885 | 258 | 3.7e-68 |
| CBJ90082.1 | Non\_Ribosomal\_peptide\_synthetase\_(-succinylbenzoate--CoA\_ligase) | BGC0001132 | Xenotetrapeptide\_biosynthetic\_gene\_cluster | 28 | 101.58061117 | 256 | 1.1e-67 |
| AHI59109.1 | locillomycin\_synthase\_B | BGC0001005 | Locillomycin\_biosynthetic\_gene\_cluster | 28 | 103.477344573 | 253 | 1.2e-66 |
| AGM16414.1 | paenibacterin\_synthetase\_C | BGC0000400 | Paenibacterin\_biosynthetic\_gene\_cluster | 30 | 88.7249736565 | 252 | 1.6e-66 |
| AAU34202.1 | mannopeptimycin\_peptide\_synthetase\_MppA | BGC0000388 | Mannopeptimycin\_biosynthetic\_gene\_cluster | 27 | 105.163329821 | 252 | 2e-66 |
| AEH41793.1 | HrmO | BGC0000374 | Hormaomycin\_biosynthetic\_gene\_cluster | 29 | 77.0284510011 | 251 | 4.5e-66 |
| AJW76709.1 | DsaG | BGC0001196 | Desotamide\_biosynthetic\_gene\_cluster | 29 | 98.6301369863 | 249 | 1.3e-65 |
| ACA97576.1 | PmxA | BGC0000408 | Polymyxin\_biosynthetic\_gene\_cluster | 31 | 67.5447839831 | 248 | 2.9e-65 |
| CDG17985.1 | Putative\_Ornithine\_racemase\_(fragment) | BGC0000464 | Xenoamicins\_biosynthetic\_gene\_cluster | 27 | 106.849315068 | 247 | 5e-65 |
| AAC06348.1 | bacitracin\_synthetase\_3 | BGC0000310 | Bacitracin\_biosynthetic\_gene\_cluster | 26 | 100.210748156 | 245 | 2.5e-64 |
| AJM89735.1 | PmxA | BGC0001192 | Colistin\_biosynthetic\_gene\_cluster | 32 | 68.177028451 | 244 | 5.5e-64 |
| AEG64696.1 | LpmB | BGC0000379 | Laspartomycin\_biosynthetic\_gene\_cluster | 29 | 87.2497365648 | 242 | 2.1e-63 |
| AEZ51516.1 | PmxA | BGC0001153 | Polymyxin\_biosynthetic\_gene\_cluster | 32 | 67.6501580611 | 241 | 4.7e-63 |
| BAH22764.1 | nonribosomal\_peptide\_synthetase | BGC0001018 | Micropeptin\_biosynthetic\_gene\_cluster | 26 | 96.9441517387 | 240 | 6.1e-63 |
| BAB69699.1 | iturin\_A\_synthetase\_B | BGC0001098 | Iturin\_biosynthetic\_gene\_cluster | 27 | 104.425711275 | 240 | 8e-63 |
| BAH43871.1 | truncated\_linear\_pentadecapeptide\_gramicidin\_synthetase\_LgrC | BGC0000367 | Gramicidin\_biosynthetic\_gene\_cluster | 27 | 88.8303477345 | 238 | 2.3e-62 |
| CAD29798.1 | peptide\_synthetase | BGC0001015 | Microcystin\_biosynthetic\_gene\_cluster | 26 | 104.214963119 | 238 | 3e-62 |
| AGM16413.1 | paenibacterin\_synthetase\_B | BGC0000400 | Paenibacterin\_biosynthetic\_gene\_cluster | 30 | 68.7038988409 | 237 | 5.2e-62 |
| AHH53507.1 | non-ribosomal\_peptide\_synthetase | BGC0000439 | Taromycin\_biosynthetic\_gene\_cluster | 29 | 91.4646996839 | 237 | 5.2e-62 |
| CAE02631.1 | surfactin\_synthetase\_B | BGC0000433 | Surfactin\_biosynthetic\_gene\_cluster | 27 | 103.371970495 | 237 | 5.2e-62 |
| ABQ96384.2 | fusaricidin\_synthetase | BGC0001152 | Fusaricidin\_biosynthetic\_gene\_cluster | 27 | 92.4130663857 | 237 | 6.7e-62 |
| CDG17980.1 | Putative\_Ornithine\_racemase\_(fragment) | BGC0000464 | Xenoamicins\_biosynthetic\_gene\_cluster | 26 | 107.165437302 | 236 | 1.5e-61 |
| ALK27914.1 | non-ribosomal\_peptide\_synthase | BGC0001233 | Feglymycin\_biosynthetic\_gene\_cluster | 31 | 66.3856691254 | 235 | 2e-61 |
| AAC06347.1 | bacitracin\_synthetase\_2 | BGC0000310 | Bacitracin\_biosynthetic\_gene\_cluster | 24 | 99.894625922 | 235 | 2e-61 |
| ACA09733.2 | fusaricidin\_synthetase | BGC0000358 | Fusaricidin\_biosynthetic\_gene\_cluster | 29 | 67.2286617492 | 233 | 9.7e-61 |
| ctg1\_orf19 |  | BGC0001013 | Meridamycin\_biosynthetic\_gene\_cluster | 27 | 94.2044257113 | 232 | 1.7e-60 |
| ABC87508.1 | NRPS\_for\_pipecolate\_incorporation | BGC0001011 | Meridamycin\_biosynthetic\_gene\_cluster | 27 | 94.2044257113 | 232 | 1.7e-60 |
| ALK27915.1 | non-ribosomal\_peptide\_synthase | BGC0001233 | Feglymycin\_biosynthetic\_gene\_cluster | 31 | 68.387776607 | 232 | 1.7e-60 |
| CAQ71828.1 | non\_ribosomal\_peptide\_synthase,\_antibiotic\_synthesis;\_contains\_3\_condensation\_domains,\_2\_AMP-acid\_ligases\_II\_domains,\_2\_PP-binding,\_Phosphopantetheine\_attachment\_site | BGC0001189 | Taiwachelin\_biosynthetic\_gene\_cluster | 32 | 67.2286617492 | 230 | 6.3e-60 |
| ABI26078.1 | OciB | BGC0000331 | Cyanopeptin\_biosynthetic\_gene\_cluster | 27 | 100.948366702 | 230 | 8.2e-60 |
| AAZ03550.1 | McnA | BGC0000332 | Cyanopeptolin\_biosynthetic\_gene\_cluster | 30 | 66.9125395153 | 230 | 1.1e-59 |
| AAO62586.1 | peptide\_sythetase | BGC0001016 | Microcystin\_biosynthetic\_gene\_cluster | 27 | 89.9894625922 | 227 | 5.3e-59 |
| CBG75492.1 | putative\_NRPS/siderophore\_biosynthesis\_protein | BGC0000423 | Scabichelin\_biosynthetic\_gene\_cluster | 27 | 107.481559536 | 227 | 9.1e-59 |
| AAF17281.1 | NosD | BGC0001028 | Nostopeptolide\_biosynthetic\_gene\_cluster | 26 | 96.7334035827 | 226 | 1.2e-58 |
| AHD05677.1 | nonribosomal\_peptide\_ligase\_subunit | BGC0000402 | Paenilarvins\_biosynthetic\_gene\_cluster | 28 | 69.1253951528 | 226 | 1.6e-58 |
| AAY93445.1 | non-ribosomal\_peptide\_synthetase\_PvdL | BGC0000413 | Pyoverdine\_biosynthetic\_gene\_cluster | 28 | 95.258166491 | 226 | 1.6e-58 |
| AGM14934.1 | xantholysin\_synthetase\_C | BGC0000463 | Xantholysin\_biosynthetic\_gene\_cluster | 28 | 90.937829294 | 226 | 1.6e-58 |
| CAD91220.1 | putative\_non-ribosomal\_peptide\_synthetase,\_modules\_1-2 | BGC0000289 | A40926\_biosynthetic\_gene\_cluster | 32 | 66.8071654373 | 226 | 1.6e-58 |
| CAQ71829.1 | non\_ribosomal\_peptide\_synthase,\_antibiotic\_synthesis;\_contains\_4\_condensation\_domains,\_3\_AMP-acid\_ligases\_II\_domains,\_3\_PP-binding,\_Phosphopantetheine\_attachment\_site\_and\_a\_putative\_thioesterase\_domain | BGC0001189 | Taiwachelin\_biosynthetic\_gene\_cluster | 27 | 95.258166491 | 225 | 2e-58 |
| BAH43870.1 | putative\_linear\_pentadecapeptide\_gramicidin\_synthetase\_LgrB | BGC0000367 | Gramicidin\_biosynthetic\_gene\_cluster | 27 | 94.7312961012 | 225 | 2.7e-58 |
| CAD91212.1 | putative\_non-ribosomal\_peptide\_synthetase,\_modules\_4-6 | BGC0000289 | A40926\_biosynthetic\_gene\_cluster | 30 | 66.9125395153 | 225 | 2.7e-58 |
| KPN93063.1 | NupA | BGC0001416 | Nunapeptin\_/\_nunamycin\_biosynthetic\_gene\_cluster | 29 | 79.5574288725 | 224 | 4.5e-58 |
| ABH06369.2 | MassC | BGC0000389 | Massetolide\_A\_biosynthetic\_gene\_cluster | 28 | 94.4151738672 | 224 | 5.9e-58 |
| AAF00960.1 | McyA | BGC0001017 | Microcystin\_biosynthetic\_gene\_cluster | 29 | 66.8071654373 | 223 | 1e-57 |
| AAO72425.1 | syringopeptin\_synthetase\_C | BGC0000438 | Syringopeptin\_biosynthetic\_gene\_cluster | 28 | 79.9789251844 | 223 | 1.3e-57 |
| AIW82284.1 | PuwG | BGC0001125 | Puwainaphycins\_biosynthetic\_gene\_cluster | 26 | 96.9441517387 | 223 | 1.3e-57 |
| AAC06346.1 | bacitracin\_synthetase\_1 | BGC0000310 | Bacitracin\_biosynthetic\_gene\_cluster | 26 | 102.845100105 | 222 | 2.2e-57 |
| AIW82283.1 | PuwF | BGC0001125 | Puwainaphycins\_biosynthetic\_gene\_cluster | 27 | 87.4604847208 | 222 | 2.2e-57 |
| ABI26077.1 | OciA | BGC0000331 | Cyanopeptin\_biosynthetic\_gene\_cluster | 30 | 66.9125395153 | 222 | 2.9e-57 |
| ABS74181.1 | BmyA | BGC0001090 | Bacillomycin\_biosynthetic\_gene\_cluster | 28 | 68.5985247629 | 221 | 3.8e-57 |
| AFH75330.1 | nonribosomal\_peptide\_synthetase | BGC0000398 | Orfamide\_biosynthetic\_gene\_cluster | 28 | 95.0474183351 | 221 | 3.8e-57 |
| AEW31022.1 | plipastatin\_synthetase | BGC0000407 | Plipastatin\_biosynthetic\_gene\_cluster | 28 | 67.4394099052 | 221 | 3.8e-57 |
| AEW31020.1 | plipastatin\_synthetase | BGC0000407 | Plipastatin\_biosynthetic\_gene\_cluster | 28 | 67.6501580611 | 221 | 3.8e-57 |
| BAC67536.1 | arthrofactin\_synthetase\_C | BGC0000305 | Arthrofactin\_biosynthetic\_gene\_cluster | 27 | 93.0453108535 | 221 | 5e-57 |
| ABV79987.1 | ApnC | BGC0000301 | Anabaenopeptin\_biosynthetic\_gene\_cluster | 28 | 67.8609062171 | 221 | 5e-57 |
| AHF21229.1 | TriE | BGC0000449 | Tridecaptin\_biosynthetic\_gene\_cluster | 32 | 67.3340358272 | 221 | 5e-57 |
| BAB69698.1 | iturin\_A\_synthetase\_A | BGC0001098 | Iturin\_biosynthetic\_gene\_cluster | 28 | 66.4910432034 | 220 | 6.5e-57 |
| KPN90376.1 | NupC | BGC0001416 | Nunapeptin\_/\_nunamycin\_biosynthetic\_gene\_cluster | 26 | 104.320337197 | 220 | 8.5e-57 |
| AAY37655.1 | Amino\_acid\_adenylation | BGC0000437 | Syringomycin\_biosynthetic\_gene\_cluster | 28 | 80.0842992624 | 220 | 1.1e-56 |
| CAA11795.1 | PCZA363.4 | BGC0000322 | Chloroeremomycin\_biosynthetic\_gene\_cluster | 31 | 66.3856691254 | 219 | 1.9e-56 |
| BAH43766.1 | tyrocidine\_synthetase\_III | BGC0000452 | Tyrocidine\_biosynthetic\_gene\_cluster | 25 | 88.3034773446 | 219 | 1.9e-56 |
| AFJ14795.1 | PlpF | BGC0000403 | Pelgipeptin\_biosynthetic\_gene\_cluster | 30 | 69.9683877766 | 218 | 2.5e-56 |
| AJI44167.1 | long-chain-fatty-acid-CoA\_ligase | BGC0001193 | Thiolutin\_biosynthetic\_gene\_cluster | 32 | 66.4910432034 | 218 | 2.5e-56 |
| AAO23333.1 | NcpA | BGC0000397 | Nostocyclopeptide\_biosynthetic\_gene\_cluster | 26 | 98.8408851423 | 218 | 3.2e-56 |
| ABV56604.1 | adenylation\_domain\_protein | BGC0000378 | Kutznerides\_biosynthetic\_gene\_cluster | 31 | 66.7017913593 | 218 | 3.2e-56 |
| AGM16412.1 | paenibacterin\_synthetase\_A | BGC0000400 | Paenibacterin\_biosynthetic\_gene\_cluster | 30 | 67.966280295 | 218 | 3.2e-56 |
| AHB82058.1 | non\_ribosomal\_peptide\_synthetase | BGC0001019 | Microsclerodermins\_biosynthetic\_gene\_cluster | 35 | 44.0463645943 | 218 | 3.2e-56 |
| CAC48361.1 | peptide\_synthetase | BGC0000311 | Balhimycin\_biosynthetic\_gene\_cluster | 31 | 66.1749209694 | 217 | 5.5e-56 |
| CAE53352.1 | non-ribosomal\_peptide\_synthetase | BGC0000440 | Teicoplanin\_biosynthetic\_gene\_cluster | 30 | 67.3340358272 | 217 | 7.2e-56 |
| AAZ03552.1 | McnC | BGC0000332 | Cyanopeptolin\_biosynthetic\_gene\_cluster | 26 | 100.52687039 | 217 | 7.2e-56 |
| ABW17377.1 | PsoC | BGC0000411 | Putisolvin\_biosynthetic\_gene\_cluster | 27 | 94.8366701791 | 217 | 7.2e-56 |
| BAB69700.1 | iturin\_A\_synthetase\_C | BGC0001098 | Iturin\_biosynthetic\_gene\_cluster | 28 | 66.2802950474 | 217 | 9.4e-56 |
| AAO23334.1 | NcpB | BGC0000397 | Nostocyclopeptide\_biosynthetic\_gene\_cluster | 26 | 93.8883034773 | 216 | 1.2e-55 |
| CAD29799.1 | microcystin\_synthetase | BGC0001015 | Microcystin\_biosynthetic\_gene\_cluster | 30 | 68.5985247629 | 216 | 1.2e-55 |
| AIE77059.1 | peptide\_synthetase | BGC0000418 | Ristocetin\_biosynthetic\_gene\_cluster | 30 | 67.966280295 | 216 | 1.6e-55 |
| AGM14933.1 | xantholysin\_synthetase\_B | BGC0000463 | Xantholysin\_biosynthetic\_gene\_cluster | 26 | 104.847207587 | 216 | 1.6e-55 |
| AEA30274.1 | peptide\_synthetase | BGC0000429 | Skyllamycin\_biosynthetic\_gene\_cluster | 30 | 66.9125395153 | 214 | 4.7e-55 |
| ACG60782.1 | NRPS(C/A/PCP/C/A/PCP) | BGC0001058 | Zorbamycin\_biosynthetic\_gene\_cluster | 33 | 66.0695468915 | 214 | 4.7e-55 |
| AEI58866.1 | peptide\_synthetase | BGC0000455 | Vancomycin\_biosynthetic\_gene\_cluster | 31 | 68.177028451 | 213 | 8e-55 |
| AEU11003.1 | NpnC | BGC0001029 | Nostophycin\_biosynthetic\_gene\_cluster | 31 | 67.7555321391 | 213 | 8e-55 |
| AGS77309.1 | NRPS\_modules\_4-6 | BGC0001178 | UK-68,597\_biosynthetic\_gene\_cluster | 30 | 69.4415173867 | 213 | 1e-54 |
| AKP45395.1 | CysG | BGC0001413 | Cystobactamide\_biosynthetic\_gene\_cluster | 26 | 101.791359326 | 213 | 1e-54 |
| CAJ21198.2 | non-ribosomal\_peptide\_synthetase | BGC0000297 | Aeruginoside\_biosynthetic\_gene\_cluster | 25 | 103.266596417 | 213 | 1.4e-54 |
| AAY37654.1 | Amino\_acid\_adenylation | BGC0000437 | Syringomycin\_biosynthetic\_gene\_cluster | 26 | 106.533192835 | 212 | 1.8e-54 |
| ADJ63842.1 | non-ribosomal\_peptide\_synthetase\_protein | BGC0000424 | Serobactins\_biosynthetic\_gene\_cluster | 30 | 65.8587987355 | 212 | 3e-54 |
| WP\_010369430.1 | Non-ribosomal\_peptide\_synthase | BGC0000314 | Bromoalterochromides\_biosynthetic\_gene\_cluster | 25 | 103.898840885 | 212 | 3e-54 |
| AEC14348.1 | nonribosomal\_peptide\_synthetase | BGC0000377 | Koranimine\_biosynthetic\_gene\_cluster | 29 | 66.9125395153 | 211 | 4e-54 |
| CCJ67639.1 | TaaD | BGC0000447 | Tolaasin\_biosynthetic\_gene\_cluster | 28 | 89.7787144362 | 211 | 5.2e-54 |
| ACZ55944.1 | non-ribosomal\_peptide\_synthetase | BGC0000302 | Anabaenopeptin\_biosynthetic\_gene\_cluster | 27 | 67.966280295 | 211 | 5.2e-54 |
| ABS74180.1 | BmyB | BGC0001090 | Bacillomycin\_biosynthetic\_gene\_cluster | 26 | 66.3856691254 | 210 | 8.8e-54 |
| AGC45618.1 | non-ribosomal\_peptide\_synthetase | BGC0001394 | Phenalamide\_biosynthetic\_gene\_cluster | 30 | 70.9167544784 | 209 | 1.5e-53 |
| CCJ67637.1 | TaaB | BGC0000447 | Tolaasin\_biosynthetic\_gene\_cluster | 29 | 79.8735511064 | 209 | 2e-53 |
| ABI26079.1 | OciC | BGC0000331 | Cyanopeptin\_biosynthetic\_gene\_cluster | 28 | 69.4415173867 | 209 | 2e-53 |
| ACA97580.1 | PmxE | BGC0000408 | Polymyxin\_biosynthetic\_gene\_cluster | 29 | 68.5985247629 | 209 | 2e-53 |
| AHZ34243.1 | CipF | BGC0001389 | Cichopeptin\_biosynthetic\_gene\_cluster | 27 | 96.1011591149 | 208 | 2.6e-53 |
| ABS74206.1 | FenD | BGC0001095 | Fengycin\_biosynthetic\_gene\_cluster | 26 | 103.793466807 | 208 | 2.6e-53 |
| AAX31559.1 | peptide\_synthetase\_3 | BGC0000336 | Daptomycin\_biosynthetic\_gene\_cluster | 28 | 92.6238145416 | 208 | 2.6e-53 |
| AIG79241.1 | Hypothetical\_protein | BGC0000419 | Ristomycin\_A\_biosynthetic\_gene\_cluster | 30 | 69.6522655427 | 208 | 3.4e-53 |
| CBJ90288.1 | Peptide\_synthetase | BGC0000416 | Rhabdopeptides\_biosynthetic\_gene\_cluster | 34 | 44.4678609062 | 208 | 4.4e-53 |
| AAF99707.2 | syringopeptin\_synthetase | BGC0000438 | Syringopeptin\_biosynthetic\_gene\_cluster | 29 | 81.875658588 | 207 | 5.7e-53 |
| BAP05597.1 | CalI | BGC0000967 | Calyculin\_biosynthetic\_gene\_cluster | 29 | 70.4952581665 | 207 | 5.7e-53 |
| BAC67535.1 | arthrofactin\_synthetase\_B | BGC0000305 | Arthrofactin\_biosynthetic\_gene\_cluster | 30 | 67.0179135933 | 207 | 5.7e-53 |
| ACA97577.1 | PmxB | BGC0000408 | Polymyxin\_biosynthetic\_gene\_cluster | 30 | 67.4394099052 | 207 | 7.5e-53 |
| AAM80537.1 | StaC | BGC0000290 | A47934\_biosynthetic\_gene\_cluster | 31 | 66.0695468915 | 207 | 7.5e-53 |
| AAZ03554.1 | McnE | BGC0000332 | Cyanopeptolin\_biosynthetic\_gene\_cluster | 28 | 69.2307692308 | 206 | 1.3e-52 |
| CAG15011.1 | peptide\_synthetase,\_module\_4-6 | BGC0000441 | Teicoplanin\_biosynthetic\_gene\_cluster | 29 | 67.3340358272 | 206 | 1.3e-52 |
| AAU39359.1 | lichenysin\_synthase\_LchAA | BGC0000381 | Lichenysin\_biosynthetic\_gene\_cluster | 29 | 68.4931506849 | 206 | 1.7e-52 |
| ABD65958.1 | nonribosomal\_peptide\_synthetase | BGC0000341 | Enduracidin\_biosynthetic\_gene\_cluster | 28 | 79.7681770285 | 205 | 2.2e-52 |
| AHH53506.1 | non-ribosomal\_peptide\_synthetase | BGC0000439 | Taromycin\_biosynthetic\_gene\_cluster | 27 | 96.9441517387 | 205 | 2.8e-52 |
| ABI22133.1 | putative\_non-ribosomal\_peptide\_synthetase | BGC0000422 | Saframycin\_A\_biosynthetic\_gene\_cluster | 29 | 66.4910432034 | 205 | 2.8e-52 |
| AGZ03651.1 | SevB | BGC0000426 | Sevadicin\_biosynthetic\_gene\_cluster | 28 | 73.2349841939 | 205 | 3.7e-52 |
| AHZ20774.1 | non-ribosomal\_peptide\_synthase | BGC0000369 | Hassallidins\_biosynthetic\_gene\_cluster | 27 | 96.2065331928 | 205 | 3.7e-52 |
| AJM89738.1 | PmxE | BGC0001192 | Colistin\_biosynthetic\_gene\_cluster | 29 | 68.8092729189 | 205 | 3.7e-52 |
| AEP18656.1 | WAPS1 | BGC0000461 | WAP-8294A2\_(lotilibcin)\_biosynthetic\_gene\_cluster | 30 | 67.7555321391 | 204 | 4.8e-52 |
| AJM89734.1 | PmxB | BGC0001192 | Colistin\_biosynthetic\_gene\_cluster | 30 | 67.966280295 | 204 | 4.8e-52 |
| AAY37653.1 | Amino\_acid\_adenylation | BGC0000437 | Syringomycin\_biosynthetic\_gene\_cluster | 26 | 108.008429926 | 204 | 6.3e-52 |
| AHD05617.1 | putative\_non-ribosomal\_peptide\_ligase\_domain\_protein | BGC0001033 | Paenilamicin\_biosynthetic\_gene\_cluster | 25 | 88.6195995785 | 204 | 6.3e-52 |
| ACU71638.1 | amino\_acid\_adenylation\_domain\_protein | BGC0001154 | Cacibiocin\_biosynthetic\_gene\_cluster | 28 | 67.2286617492 | 203 | 8.3e-52 |
| AHH53508.1 | non-ribosomal\_peptide\_synthetase | BGC0000439 | Taromycin\_biosynthetic\_gene\_cluster | 29 | 66.7017913593 | 203 | 8.3e-52 |
| AEZ51520.1 | PmxE | BGC0001153 | Polymyxin\_biosynthetic\_gene\_cluster | 29 | 68.4931506849 | 203 | 8.3e-52 |
| AEZ51517.1 | PmxB | BGC0001153 | Polymyxin\_biosynthetic\_gene\_cluster | 28 | 76.2908324552 | 203 | 8.3e-52 |
| BAE98155.1 | putative\_non-ribosomal\_peptide\_synthetase | BGC0000339 | Echinomycin\_biosynthetic\_gene\_cluster | 28 | 78.5036880927 | 203 | 8.3e-52 |
| ADH04679.1 | non-ribosomal\_peptide\_synthetase | BGC0001344 | Tubulysin\_biosynthetic\_gene\_cluster | 28 | 105.900948367 | 203 | 1.1e-51 |
| AAF15891.2 | NosA | BGC0001028 | Nostopeptolide\_biosynthetic\_gene\_cluster | 30 | 68.7038988409 | 203 | 1.4e-51 |
| AHD05614.1 | putative\_non-ribosomal\_peptide\_ligase/\_polyketide\_synthase\_hybrid | BGC0001033 | Paenilamicin\_biosynthetic\_gene\_cluster | 28 | 65.8587987355 | 202 | 1.8e-51 |
| ABD14711.1 | CesA | BGC0000320 | Cereulide\_biosynthetic\_gene\_cluster | 29 | 67.7555321391 | 202 | 1.8e-51 |
| AHZ34233.1 | CifB | BGC0000323 | Cichofactin\_biosynthetic\_gene\_cluster | 29 | 67.2286617492 | 202 | 1.8e-51 |
| AGE25480.1 | amino\_acid\_adenylation\_protein | BGC0001208\_c2 | Poaeamide\_biosynthetic\_gene\_cluster | 30 | 68.5985247629 | 201 | 3.1e-51 |
| CAM02313.1 | putative\_non-ribosomal\_peptide\_synthetase | BGC0000349 | Erythrochelin\_biosynthetic\_gene\_cluster | 36 | 44.9947312961 | 201 | 3.1e-51 |
| BAH43765.1 | tyrocidine\_synthetase\_II | BGC0000452 | Tyrocidine\_biosynthetic\_gene\_cluster | 32 | 45.8377239199 | 201 | 4.1e-51 |
| AHB38511.1 | EpnJ | BGC0000345 | Eponemycin\_biosynthetic\_gene\_cluster | 31 | 69.8630136986 | 201 | 4.1e-51 |
| AAM80539.1 | StaA | BGC0000290 | A47934\_biosynthetic\_gene\_cluster | 29 | 65.8587987355 | 201 | 5.4e-51 |
| AEU11006.1 | NpnB | BGC0001029 | Nostophycin\_biosynthetic\_gene\_cluster | 31 | 67.0179135933 | 201 | 5.4e-51 |
| ABS74179.1 | BmyC | BGC0001090 | Bacillomycin\_biosynthetic\_gene\_cluster | 29 | 67.2286617492 | 201 | 5.4e-51 |
| AAF17280.1 | NosC | BGC0001028 | Nostopeptolide\_biosynthetic\_gene\_cluster | 25 | 98.4193888303 | 201 | 5.4e-51 |
| CAK15814.1 | putative\_non-ribosomal\_peptide\_synthetase,\_terminal\_component | BGC0000344 | Entolysin\_biosynthetic\_gene\_cluster | 27 | 104.425711275 | 200 | 7e-51 |
| AAO72424.1 | syringopeptin\_synthetase\_B | BGC0000438 | Syringopeptin\_biosynthetic\_gene\_cluster | 29 | 67.2286617492 | 200 | 9.1e-51 |
| CAE02630.1 | surfactin\_synthetase\_A | BGC0000433 | Surfactin\_biosynthetic\_gene\_cluster | 28 | 70.1791359326 | 200 | 9.1e-51 |
| ABD65957.1 | nonribosomal\_peptide\_synthetase | BGC0000341 | Enduracidin\_biosynthetic\_gene\_cluster | 28 | 88.7249736565 | 199 | 1.6e-50 |
| KPN93064.1 | NupB | BGC0001416 | Nunapeptin\_/\_nunamycin\_biosynthetic\_gene\_cluster | 30 | 66.5964172813 | 199 | 1.6e-50 |
| ABG94125.1 | non-ribosomal\_peptide\_synthetase | BGC0000417 | Rhodochelin\_biosynthetic\_gene\_cluster | 28 | 66.4910432034 | 199 | 2e-50 |
| AFH75329.1 | nonribosomal\_peptide\_synthetase | BGC0000398 | Orfamide\_biosynthetic\_gene\_cluster | 29 | 67.3340358272 | 198 | 2.7e-50 |
| CCJ67648.1 | JagD | BGC0001127 | Jagaricin\_biosynthetic\_gene\_cluster | 31 | 69.5468914647 | 198 | 2.7e-50 |
| CCJ67647.1 | JagC | BGC0001127 | Jagaricin\_biosynthetic\_gene\_cluster | 26 | 93.5721812434 | 198 | 2.7e-50 |
| AID65225.1 | nonribosomal\_peptide\_synthetase | BGC0000335 | Cystomanamides\_biosynthetic\_gene\_cluster | 30 | 67.7555321391 | 198 | 2.7e-50 |
| AAF67501.2 | NovH | BGC0000834 | Novobiocin\_biosynthetic\_gene\_cluster | 33 | 45.626975764 | 198 | 4.5e-50 |
| CCM44336.1 | putative\_nonribosomal\_peptide\_synthetase | BGC0001056 | Zeamine\_biosynthetic\_gene\_cluster | 34 | 45.9430979979 | 198 | 4.5e-50 |
| AIE77057.1 | peptide\_synthetase | BGC0000418 | Ristocetin\_biosynthetic\_gene\_cluster | 29 | 67.6501580611 | 197 | 5.9e-50 |
| ALV86867.1 | Tlo21 | BGC0001406 | Telomycin\_biosynthetic\_gene\_cluster | 35 | 44.2571127503 | 197 | 5.9e-50 |
| AGC09528.1 | NRPS | BGC0001183 | Lobophorin\_biosynthetic\_gene\_cluster | 27 | 101.369863014 | 196 | 1e-49 |
| ADA82585.1 | hybrid\_trans-AT\_polyketide\_synthase\_-\_nonribosomal\_peptide\_synthetase | BGC0001110 | Psymberin\_biosynthetic\_gene\_cluster | 27 | 69.5468914647 | 196 | 1e-49 |
| AKP45399.1 | CysK | BGC0001413 | Cystobactamide\_biosynthetic\_gene\_cluster | 26 | 102.212855638 | 196 | 1.3e-49 |
| AHZ34238.1 | CipA | BGC0001389 | Cichopeptin\_biosynthetic\_gene\_cluster | 26 | 104.109589041 | 196 | 1.3e-49 |
| BAO98812.1 | putative\_nonribosomal\_peptide\_synthetase | BGC0001002 | Jawsamycin\_biosynthetic\_gene\_cluster | 33 | 43.5194942044 | 196 | 1.3e-49 |
| AEG64697.1 | LpmC | BGC0000379 | Laspartomycin\_biosynthetic\_gene\_cluster | 35 | 44.0463645943 | 196 | 1.3e-49 |
| ALG65314.1 | Cal22 | BGC0001297 | WS9326\_biosynthetic\_gene\_cluster | 29 | 67.6501580611 | 196 | 1.7e-49 |
| AAX31557.1 | peptide\_synthetase\_1 | BGC0000336 | Daptomycin\_biosynthetic\_gene\_cluster | 30 | 67.3340358272 | 195 | 2.2e-49 |
| AJW65406.1 | nonribosomal\_peptide\_synthetase | BGC0001195 | Nocardiopsin\_biosynthetic\_gene\_cluster | 31 | 64.3835616438 | 195 | 2.2e-49 |
| AHD05678.1 | nonribosomal\_peptide\_ligase\_subunit | BGC0000402 | Paenilarvins\_biosynthetic\_gene\_cluster | 31 | 44.5732349842 | 195 | 2.9e-49 |
| AHD05679.1 | putative\_non-ribosomal\_peptide\_ligase/\_polyketide\_synthase\_hybrid | BGC0000402 | Paenilarvins\_biosynthetic\_gene\_cluster | 31 | 44.5732349842 | 195 | 2.9e-49 |
| WP\_051700112.1 | non-ribosomal\_peptide\_synthetase | BGC0001368 | Tambromycin\_biosynthetic\_gene\_cluster | 28 | 72.602739726 | 194 | 5e-49 |
| CCA29203.1 | non-ribosomal\_peptide\_synthetase/polyketide\_synthase | BGC0000955 | Althiomycin\_biosynthetic\_gene\_cluster | 29 | 68.5985247629 | 194 | 6.5e-49 |
| CAJ46692.1 | non\_ribosomal\_peptide\_synthase | BGC0000969 | Chondramide\_biosynthetic\_gene\_cluster | 28 | 63.2244467861 | 194 | 6.5e-49 |
| CAA11794.1 | PCZA363.3 | BGC0000322 | Chloroeremomycin\_biosynthetic\_gene\_cluster | 35 | 46.3645943098 | 193 | 8.5e-49 |
| AHZ34239.1 | CipB | BGC0001389 | Cichopeptin\_biosynthetic\_gene\_cluster | 26 | 104.847207587 | 193 | 8.5e-49 |
| ABP57749.1 | DepE | BGC0000993 | FK228\_biosynthetic\_gene\_cluster | 28 | 91.2539515279 | 193 | 1.1e-48 |
| ABS90476.1 | NRPS | BGC0001106 | Oxazolomycin\_biosynthetic\_gene\_cluster | 30 | 67.4394099052 | 193 | 1.1e-48 |
| ABP57748.1 | DepD | BGC0000993 | FK228\_biosynthetic\_gene\_cluster | 29 | 68.9146469968 | 193 | 1.1e-48 |
| BAO84866.1 | putative\_non-ribosomal\_peptide\_synthetase | BGC0000414 | Quinocarcin\_biosynthetic\_gene\_cluster | 28 | 67.8609062171 | 193 | 1.5e-48 |
| WP\_020635019.1 | hypothetical\_protein | BGC0001211 | Albachelin\_biosynthetic\_gene\_cluster | 34 | 43.6248682824 | 193 | 1.5e-48 |
| AEH59100.1 | amino\_acid\_adenylation\_domain-containing\_protein/NRPS | BGC0000385 | Lysobactin\_biosynthetic\_gene\_cluster | 28 | 67.5447839831 | 192 | 1.9e-48 |
| AAF08796.1 | MycB | BGC0001103 | Mycosubtilin\_biosynthetic\_gene\_cluster | 24 | 100.632244468 | 192 | 1.9e-48 |
| AHD05618.1 | putative\_non-ribosomal\_peptide\_ligase\_domain\_protein | BGC0001033 | Paenilamicin\_biosynthetic\_gene\_cluster | 27 | 68.9146469968 | 192 | 2.5e-48 |
| ctg1\_orf6 |  | BGC0001109 | Pederin\_biosynthetic\_gene\_cluster | 28 | 67.1232876712 | 192 | 2.5e-48 |
| AAS47564.1 | mixed\_type\_I\_polyketide\_synthase/nonribosomal\_peptide\_synthetase | BGC0001108 | Pederin\_biosynthetic\_gene\_cluster | 28 | 67.1232876712 | 192 | 2.5e-48 |
| AAZ23077.1 | peptide\_synthetase | BGC0000291 | A54145\_biosynthetic\_gene\_cluster | 30 | 70.2845100105 | 191 | 3.2e-48 |
| CDG17986.1 | Non-ribosomal\_peptide\_synthetase | BGC0000464 | Xenoamicins\_biosynthetic\_gene\_cluster | 25 | 107.165437302 | 191 | 3.2e-48 |
| ADA69239.2 | trans-AT\_hybrid\_polyketide\_synthase-NRPS | BGC0001071 | Nosperin\_biosynthetic\_gene\_cluster | 33 | 45.416227608 | 191 | 3.2e-48 |
| ACG60773.1 | NRPS(C/A/PCP) | BGC0001058 | Zorbamycin\_biosynthetic\_gene\_cluster | 29 | 67.4394099052 | 191 | 3.2e-48 |
| CAJ77715.1 | Mps1\_protein | BGC0000364 | Glycopeptidolipid\_biosynthetic\_gene\_cluster | 29 | 69.2307692308 | 191 | 4.2e-48 |
| AAF08795.1 | MycA | BGC0001103 | Mycosubtilin\_biosynthetic\_gene\_cluster | 32 | 43.0979978925 | 191 | 4.2e-48 |
| AAC32047.1 | FxbB | BGC0000351 | Exochelin\_biosynthetic\_gene\_cluster | 29 | 68.8092729189 | 191 | 5.5e-48 |
| ABD65956.1 | nonribosomal\_peptide\_synthetase | BGC0000341 | Enduracidin\_biosynthetic\_gene\_cluster | 30 | 67.7555321391 | 191 | 5.5e-48 |
| AIG79243.1 | Hypothetical\_protein | BGC0000419 | Ristomycin\_A\_biosynthetic\_gene\_cluster | 29 | 67.8609062171 | 191 | 5.5e-48 |
| AAM80538.1 | StaB | BGC0000290 | A47934\_biosynthetic\_gene\_cluster | 28 | 66.0695468915 | 190 | 7.2e-48 |
| AHZ20781.1 | non-ribosomal\_peptide\_synthase | BGC0000369 | Hassallidins\_biosynthetic\_gene\_cluster | 27 | 77.133825079 | 190 | 7.2e-48 |
| AAU34203.1 | mannopeptimycin\_peptide\_synthetase\_MppB | BGC0000388 | Mannopeptimycin\_biosynthetic\_gene\_cluster | 33 | 45.416227608 | 190 | 9.5e-48 |
| ABP53497.1 | amino\_acid\_adenylation\_domain | BGC0001041 | Salinosporamide\_biosynthetic\_gene\_cluster | 29 | 64.3835616438 | 190 | 1.2e-47 |
| ABP73646.1 | SalB | BGC0000145 | Salinosporamide\_A\_biosynthetic\_gene\_cluster | 29 | 64.3835616438 | 190 | 1.2e-47 |
| ABM21571.1 | CrpC | BGC0000975 | Cryptophycin\_biosynthetic\_gene\_cluster | 34 | 45.31085353 | 190 | 1.2e-47 |
| ABL74945.1 | TlmIV | BGC0001048 | Tallysomycin\_biosynthetic\_gene\_cluster | 25 | 93.3614330875 | 190 | 1.2e-47 |
| AHH25592.1 | NRPS | BGC0000957 | Ansatrienin\_(mycotrienin)\_biosynthetic\_gene\_cluster | 30 | 65.3319283456 | 189 | 1.6e-47 |
| ALV82384.1 | CDA\_peptide\_synthetase\_II | BGC0001370 | Lipopeptide\_8D1-1\_/\_lipopeptide\_8D1-2\_biosynthetic\_gene\_cluster | 30 | 68.4931506849 | 188 | 2.8e-47 |
| CAA11773.1 | PCZA361.18 | BGC0000322\_c2 | Chloroeremomycin\_biosynthetic\_gene\_cluster | 33 | 42.5711275026 | 188 | 3.6e-47 |
| ABI22132.1 | putative\_non-ribosomal\_peptide\_synthetase | BGC0000422 | Saframycin\_A\_biosynthetic\_gene\_cluster | 32 | 67.3340358272 | 188 | 3.6e-47 |
| AAN65224.1 | peptide\_synthetase-like\_protein | BGC0000832 | Clorobiocin\_biosynthetic\_gene\_cluster | 30 | 51.1064278188 | 188 | 4.7e-47 |
| ABF87031.1 | non-ribosomal\_peptide\_synthetase/polyketide\_synthase | BGC0000393 | Myxoprincomide\_biosynthetic\_gene\_cluster | 30 | 71.7597471022 | 187 | 6.1e-47 |
| AFH75321.1 | nonribosomal\_peptide\_synthetase | BGC0000425 | Sessilin\_biosynthetic\_gene\_cluster | 35 | 44.4678609062 | 187 | 6.1e-47 |
| AEA30273.1 | peptide\_synthetase | BGC0000429 | Skyllamycin\_biosynthetic\_gene\_cluster | 37 | 43.5194942044 | 187 | 6.1e-47 |
| ADD82940.1 | Bat2 | BGC0001099 | Kalimantacin\_/\_batumin\_biosynthetic\_gene\_cluster | 29 | 64.3835616438 | 187 | 6.1e-47 |
| ALK27916.1 | non-ribosomal\_peptide\_synthase | BGC0001233 | Feglymycin\_biosynthetic\_gene\_cluster | 33 | 44.9947312961 | 186 | 1e-46 |
| ACU36654.1 | amino\_acid\_adenylation\_domain\_protein | BGC0000392 | Mirubactin\_biosynthetic\_gene\_cluster | 35 | 43.6248682824 | 186 | 1e-46 |
| AHI59110.1 | locillomycin\_synthase\_C | BGC0001005 | Locillomycin\_biosynthetic\_gene\_cluster | 26 | 72.602739726 | 186 | 1e-46 |
| CAC48360.1 | peptide\_synthetase | BGC0000311 | Balhimycin\_biosynthetic\_gene\_cluster | 35 | 45.2054794521 | 186 | 1.4e-46 |
| CCJ67646.1 | JagB | BGC0001127 | Jagaricin\_biosynthetic\_gene\_cluster | 34 | 43.3087460485 | 186 | 1.4e-46 |
| AGE11898.1 | nonribosomal\_peptide\_synthetase | BGC0000366 | Gobichelin\_biosynthetic\_gene\_cluster | 35 | 44.7839831401 | 186 | 1.4e-46 |
| AEI58865.1 | peptide\_synthetase | BGC0000455 | Vancomycin\_biosynthetic\_gene\_cluster | 33 | 43.8356164384 | 186 | 1.4e-46 |
| AAM80536.1 | StaD | BGC0000290 | A47934\_biosynthetic\_gene\_cluster | 29 | 67.6501580611 | 185 | 3e-46 |
| CBZ42146.1 | putative\_non-ribosomal\_peptide\_synthetase | BGC0001117 | Himastatin\_biosynthetic\_gene\_cluster | 33 | 43.5194942044 | 185 | 4e-46 |
| AAN85512.1 | nonribosomal\_peptide\_synthetase | BGC0001101 | Leinamycin\_biosynthetic\_gene\_cluster | 29 | 72.2866174921 | 185 | 4e-46 |
| AAK81827.1 | peptide\_synthetase | BGC0000326 | Complestatin\_biosynthetic\_gene\_cluster | 28 | 69.3361433087 | 185 | 4e-46 |
| CAB38517.1 | CDA\_peptide\_synthetase\_II | BGC0000315 | Calcium-dependent\_antibiotic\_biosynthetic\_gene\_cluster | 26 | 89.2518440464 | 184 | 6.8e-46 |
| AAP92497.1 | nonribosomal\_peptide\_synthetase | BGC0000458 | Viomycin\_biosynthetic\_gene\_cluster | 29 | 90.832455216 | 184 | 6.8e-46 |
| CAJ14039.1 | peptide\_synthetase\_PhsC | BGC0000406 | Phosphinothricin\_biosynthetic\_gene\_cluster | 33 | 44.3624868282 | 183 | 8.8e-46 |
| AAK81824.1 | peptide\_synthetase | BGC0000326 | Complestatin\_biosynthetic\_gene\_cluster | 35 | 44.8893572181 | 183 | 8.8e-46 |
| ABW00331.1 | amino\_acid\_adenylation\_domain | BGC0000333 | Cyclomarin\_biosynthetic\_gene\_cluster | 33 | 43.2033719705 | 183 | 8.8e-46 |
| ALV82388.1 | CDA\_peptide\_synthetase\_III | BGC0001370 | Lipopeptide\_8D1-1\_/\_lipopeptide\_8D1-2\_biosynthetic\_gene\_cluster | 34 | 45.1001053741 | 183 | 1.2e-45 |
| CAD55498.1 | CDA\_peptide\_synthetase\_III | BGC0000315 | Calcium-dependent\_antibiotic\_biosynthetic\_gene\_cluster | 35 | 45.626975764 | 183 | 1.2e-45 |
| AAS47562.1 | mixed\_type\_I\_polyketide\_synthase\_-\_peptide\_synthetase | BGC0001108 | Pederin\_biosynthetic\_gene\_cluster | 30 | 70.4952581665 | 183 | 1.5e-45 |
| ctg1\_orf8 |  | BGC0001109 | Pederin\_biosynthetic\_gene\_cluster | 30 | 70.4952581665 | 183 | 1.5e-45 |
| CAE52334.1 | non-ribosomal\_peptide\_synthase | BGC0001088 | Albicidin\_biosynthetic\_gene\_cluster | 25 | 101.369863014 | 183 | 1.5e-45 |
| BAH22765.1 | nonribosomal\_peptide\_synthetase | BGC0001018 | Micropeptin\_biosynthetic\_gene\_cluster | 32 | 47.2075869336 | 183 | 1.5e-45 |
| CBZ42140.1 | non-ribosomal\_peptide\_synthetase | BGC0001117 | Himastatin\_biosynthetic\_gene\_cluster | 29 | 64.1728134879 | 182 | 2e-45 |
| CBD77746.1 | non-ribosomal\_peptide\_synthetase/polyketide\_synthase | BGC0000974 | Crocacin\_biosynthetic\_gene\_cluster | 29 | 66.1749209694 | 182 | 2e-45 |
| AHI59108.1 | locillomycin\_synthase\_A | BGC0001005 | Locillomycin\_biosynthetic\_gene\_cluster | 28 | 69.5468914647 | 182 | 2.6e-45 |
| AHD05615.1 | putative\_non-ribosomal\_peptide\_ligase/\_polyketide\_synthase\_hybrid | BGC0001033 | Paenilamicin\_biosynthetic\_gene\_cluster | 26 | 67.4394099052 | 181 | 4.4e-45 |
| ACJ04424.1 | aureobasidin\_A1\_biosynthesis\_complex | BGC0000307 | Aureobasidin\_A1\_biosynthetic\_gene\_cluster | 29 | 68.7038988409 | 180 | 7.5e-45 |
| AHZ20773.1 | non-ribosomal\_peptide\_synthase | BGC0000369 | Hassallidins\_biosynthetic\_gene\_cluster | 27 | 65.2265542677 | 180 | 9.8e-45 |
| AFH75320.1 | nonribosomal\_peptide\_synthetase | BGC0000425 | Sessilin\_biosynthetic\_gene\_cluster | 34 | 44.2571127503 | 179 | 1.7e-44 |
| CDG12865.1 | non-ribosomal\_peptide\_synthetase | BGC0001415 | Althiomycin\_biosynthetic\_gene\_cluster | 25 | 78.5036880927 | 179 | 1.7e-44 |
| AAG29780.1 | acyl-CoA\_synthetase | BGC0000833 | Coumermycin\_A1\_biosynthetic\_gene\_cluster | 31 | 51.1064278188 | 178 | 3.7e-44 |
| AAP92505.1 | nonribosomal\_peptide\_synthetase | BGC0000458 | Viomycin\_biosynthetic\_gene\_cluster | 28 | 68.8092729189 | 178 | 3.7e-44 |
| EFE73313.1 | nonribosomal\_peptide\_synthetase | BGC0000431 | Stenothricin\_biosynthetic\_gene\_cluster | 33 | 45.416227608 | 178 | 4.9e-44 |
| AHZ34242.1 | CipE | BGC0001389 | Cichopeptin\_biosynthetic\_gene\_cluster | 27 | 81.981032666 | 178 | 4.9e-44 |
| CCE88377.1 | non-ribosomal\_peptide\_synthetase/polyketide\_synthase | BGC0001034 | Pellasoren\_biosynthetic\_gene\_cluster | 35 | 44.2571127503 | 177 | 6.3e-44 |
| AHB82069.1 | non\_ribosomal\_peptide\_synthetase | BGC0001231 | Microsclerodermins\_biosynthetic\_gene\_cluster | 32 | 46.4699683878 | 177 | 6.3e-44 |
| AEW31015.1 | plipastatin\_synthetase | BGC0000407 | Plipastatin\_biosynthetic\_gene\_cluster | 28 | 70.4952581665 | 177 | 6.3e-44 |
| AFJ23826.1 | WLIP\_synthetase\_C | BGC0000462\_c2 | WLIP\_biosynthetic\_gene\_cluster | 29 | 68.387776607 | 177 | 8.3e-44 |
| ABV56585.1 | KtzE | BGC0000378 | Kutznerides\_biosynthetic\_gene\_cluster | 27 | 69.5468914647 | 177 | 8.3e-44 |
| AAZ23075.1 | peptide\_synthetase | BGC0000291 | A54145\_biosynthetic\_gene\_cluster | 34 | 45.7323498419 | 177 | 8.3e-44 |
| AAZ55900.1 | non-ribosomal\_peptide\_synthase:Amino\_acid\_adenylation | BGC0000359 | Fuscachelin\_biosynthetic\_gene\_cluster | 29 | 68.071654373 | 176 | 1.1e-43 |
| AAU39361.1 | lichenysin\_synthase\_LchAC | BGC0000381 | Lichenysin\_biosynthetic\_gene\_cluster | 31 | 44.0463645943 | 176 | 1.4e-43 |
| AGE26255.1 | putative\_non-ribosomal\_peptide\_synthetase | BGC0001208 | Poaeamide\_biosynthetic\_gene\_cluster | 32 | 44.2571127503 | 176 | 1.8e-43 |
| CAJ34381.1 | NRPS\_protein | BGC0000445 | Thiocoraline\_biosynthetic\_gene\_cluster | 33 | 44.7839831401 | 175 | 2.4e-43 |
| ABV56588.1 | KtzH | BGC0000378 | Kutznerides\_biosynthetic\_gene\_cluster | 32 | 42.4657534247 | 175 | 2.4e-43 |
| AOA33122.1 | nonribosomal\_peptide\_synthetase | BGC0001346 | Bananamides\_biosynthetic\_gene\_cluster | 32 | 43.8356164384 | 175 | 2.4e-43 |
| AAY32966.1 | DszC | BGC0001093 | Disorazole\_biosynthetic\_gene\_cluster | 28 | 67.6501580611 | 175 | 3.1e-43 |
| CAB53322.1 | putative\_peptide\_synthetase | BGC0000325 | Coelichelin\_biosynthetic\_gene\_cluster | 32 | 45.31085353 | 175 | 4.1e-43 |
| AIW82282.1 | PuwE | BGC0001125 | Puwainaphycins\_biosynthetic\_gene\_cluster | 27 | 70.3898840885 | 174 | 5.4e-43 |
| ABJ97436.1 | MerP | BGC0001012 | Meridamycin\_biosynthetic\_gene\_cluster | 27 | 78.6090621707 | 174 | 5.4e-43 |
| AGN74885.1 | nonribosomal\_peptide\_synthetase | BGC0000459 | Griseoviridin\_/\_viridogrisein\_biosynthetic\_gene\_cluster | 27 | 103.582718651 | 174 | 5.4e-43 |
| WP\_010369428.1 | phenylalanine\_racemase | BGC0000314 | Bromoalterochromides\_biosynthetic\_gene\_cluster | 29 | 65.5426765016 | 174 | 7e-43 |
| ABW17375.1 | PsoA | BGC0000411 | Putisolvin\_biosynthetic\_gene\_cluster | 32 | 45.2054794521 | 174 | 7e-43 |
| AEC14346.1 | nonribosomal\_peptide\_synthetase | BGC0000377 | Koranimine\_biosynthetic\_gene\_cluster | 27 | 64.9104320337 | 174 | 7e-43 |
| ANQ43349.1 | non-ribosomal\_peptide\_synthetase | BGC0001347\_c2 | Poaeamide\_B\_biosynthetic\_gene\_cluster | 32 | 45.1001053741 | 173 | 9.2e-43 |
| AED90004.1 | non-ribosomal\_peptide\_synthetase\_ThaC1 | BGC0000443 | Thanamycin\_biosynthetic\_gene\_cluster | 32 | 46.2592202318 | 173 | 9.2e-43 |
| CAJ77695.1 | MPS1\_protein | BGC0000363 | Glycopeptidolipid\_biosynthetic\_gene\_cluster | 34 | 44.5732349842 | 173 | 9.2e-43 |
| CAC17499.1 | putative\_non-ribosomal\_peptide\_synthase | BGC0000324 | Coelibactin\_biosynthetic\_gene\_cluster | 29 | 67.4394099052 | 173 | 9.2e-43 |
| ANQ43348.1 | non-ribosomal\_peptide\_synthetase | BGC0001347\_c2 | Poaeamide\_B\_biosynthetic\_gene\_cluster | 31 | 43.8356164384 | 172 | 2e-42 |
| sln6 | nonribosomal\_peptide\_synthetase | BGC0001230 | Salinamides\_biosynthetic\_gene\_cluster | 28 | 69.0200210748 | 172 | 2e-42 |
| BAH22763.1 | nonribosomal\_peptide\_synthetase | BGC0001018 | Micropeptin\_biosynthetic\_gene\_cluster | 27 | 72.3919915701 | 172 | 2e-42 |
| AAS92545.1 | SirP | BGC0001044 | Sirodesmin\_biosynthetic\_gene\_cluster | 31 | 48.2613277134 | 172 | 2e-42 |
| CAY48788.1 | putative\_non-ribosomal\_peptide\_synthetase | BGC0001312 | Viscosin\_biosynthetic\_gene\_cluster | 31 | 44.5732349842 | 172 | 2.7e-42 |
| BAD55612.1 | non-ribosomal\_peptide\_synthetase | BGC0001027 | Nocobactin\_NA\_biosynthetic\_gene\_cluster | 34 | 45.626975764 | 171 | 3.5e-42 |
| BAP16699.1 | nonribosomal\_peptide\_synthetase | BGC0000376 | JBIR-34,\_JBIR-35\_biosynthetic\_gene\_cluster | 28 | 68.9146469968 | 171 | 3.5e-42 |
| CCJ67638.1 | TaaC | BGC0000447 | Tolaasin\_biosynthetic\_gene\_cluster | 33 | 44.4678609062 | 171 | 3.5e-42 |
| BAO84868.1 | putative\_non-ribosomal\_peptide\_synthetase | BGC0000414 | Quinocarcin\_biosynthetic\_gene\_cluster | 30 | 57.7449947313 | 171 | 4.5e-42 |
| ABD65960.1 | nonribosomal\_peptide\_synthetase | BGC0000341 | Enduracidin\_biosynthetic\_gene\_cluster | 29 | 67.8609062171 | 171 | 4.5e-42 |
| CAJ46691.1 | non\_ribosomal\_peptide\_synthase | BGC0000969 | Chondramide\_biosynthetic\_gene\_cluster | 26 | 66.7017913593 | 171 | 4.5e-42 |
| ACM79812.1 | ZmaQ | BGC0001059 | Zwittermycin\_A\_biosynthetic\_gene\_cluster | 30 | 45.1001053741 | 171 | 5.9e-42 |
| AAT09804.1 | NocA | BGC0000395 | Nocardicin\_A\_biosynthetic\_gene\_cluster | 32 | 44.8893572181 | 170 | 7.8e-42 |
| CAJ45639.1 | vanchrobactin\_non\_ribosomal\_peptide\_synthetase | BGC0000454 | Vanchrobactin\_biosynthetic\_gene\_cluster | 26 | 68.177028451 | 170 | 1.3e-41 |
| ALV86868.1 | Tlo22 | BGC0001406 | Telomycin\_biosynthetic\_gene\_cluster | 28 | 70.0737618546 | 170 | 1.3e-41 |
| AED90003.1 | non-ribosomal\_peptide\_synthetase\_ThaB | BGC0000443 | Thanamycin\_biosynthetic\_gene\_cluster | 31 | 44.9947312961 | 170 | 1.3e-41 |
| BAP05589.1 | CalA | BGC0000967 | Calyculin\_biosynthetic\_gene\_cluster | 30 | 45.1001053741 | 169 | 1.7e-41 |
| AAU39360.1 | lichenysin\_synthase\_LchAB | BGC0000381 | Lichenysin\_biosynthetic\_gene\_cluster | 29 | 46.0484720759 | 169 | 2.3e-41 |
| BAH43869.1 | linear\_pentadecapeptide\_gramicidin\_synthetase\_LgrA | BGC0000367 | Gramicidin\_biosynthetic\_gene\_cluster | 31 | 44.9947312961 | 169 | 2.3e-41 |
| AAY91421.3 | non-ribosomal\_peptide\_synthetase\_OfaC | BGC0000399 | Orfamide\_biosynthetic\_gene\_cluster | 32 | 44.1517386723 | 169 | 2.3e-41 |
| EFL06865.1 | hypothetical\_protein | BGC0000300 | Amychelin\_biosynthetic\_gene\_cluster | 34 | 44.0463645943 | 169 | 2.3e-41 |
| AEW31021.1 | plipastatin\_synthetase | BGC0000407 | Plipastatin\_biosynthetic\_gene\_cluster | 32 | 44.3624868282 | 169 | 2.3e-41 |
| CAJ77716.1 | Mps2\_protein | BGC0000364 | Glycopeptidolipid\_biosynthetic\_gene\_cluster | 31 | 44.9947312961 | 168 | 2.9e-41 |
| ABL74939.1 | TlmVII | BGC0001048 | Tallysomycin\_biosynthetic\_gene\_cluster | 29 | 64.3835616438 | 168 | 2.9e-41 |
| CAG15010.1 | peptide\_synthetase,\_module\_3 | BGC0000441 | Teicoplanin\_biosynthetic\_gene\_cluster | 27 | 67.1232876712 | 168 | 3.8e-41 |
| CBL93718.1 | NRPS\_didomain\_PCP-C | BGC0000360 | GE81112\_biosynthetic\_gene\_cluster | 27 | 69.7576396207 | 168 | 3.8e-41 |
| AGD80616.1 | amino\_acid\_adenylation\_domain-containing\_protein | BGC0000394 | Naphthyridinomycin\_biosynthetic\_gene\_cluster | 33 | 44.6786090622 | 168 | 3.8e-41 |
| CBW45648.1 | PI\_synthetase\_2 | BGC0000952 | Pristinamycin\_biosynthetic\_gene\_cluster | 33 | 45.2054794521 | 168 | 5e-41 |
| ACZ55945.1 | non-ribosomal\_peptide\_synthetase | BGC0000302 | Anabaenopeptin\_biosynthetic\_gene\_cluster | 31 | 45.521601686 | 167 | 6.6e-41 |
| AAD44234.1 | PstB | BGC0000362 | Glycopeptidolipid\_biosynthetic\_gene\_cluster | 32 | 44.1517386723 | 167 | 6.6e-41 |
| AAF00961.1 | McyB | BGC0001017 | Microcystin\_biosynthetic\_gene\_cluster | 30 | 44.7839831401 | 167 | 6.6e-41 |
| ABX37382.1 | amino\_acid\_adenylation\_domain\_protein | BGC0000984 | Delftibactin\_biosynthetic\_gene\_cluster | 32 | 44.0463645943 | 167 | 8.6e-41 |
| AAZ23076.1 | peptide\_synthetase | BGC0000291 | A54145\_biosynthetic\_gene\_cluster | 32 | 46.3645943098 | 167 | 8.6e-41 |
| CAM59606.1 | non-ribosomal\_peptide\_synthetase | BGC0000297 | Aeruginoside\_biosynthetic\_gene\_cluster | 32 | 45.1001053741 | 167 | 8.6e-41 |
| AGE25479.1 | putative\_non-ribosomal\_peptide\_synthetase | BGC0001208\_c2 | Poaeamide\_biosynthetic\_gene\_cluster | 30 | 44.0463645943 | 167 | 8.6e-41 |
| CAJ77696.1 | MPS2\_protein | BGC0000363 | Glycopeptidolipid\_biosynthetic\_gene\_cluster | 31 | 45.1001053741 | 166 | 1.1e-40 |
| CAE02633.1 | surfactin\_synthetase\_C | BGC0000433 | Surfactin\_biosynthetic\_gene\_cluster | 30 | 44.4678609062 | 166 | 1.1e-40 |
| AFH75322.1 | nonribosomal\_peptide\_synthetase | BGC0000425 | Sessilin\_biosynthetic\_gene\_cluster | 32 | 44.1517386723 | 166 | 1.9e-40 |
| CDG17981.1 | Non-ribosomal\_peptide\_synthetase | BGC0000464 | Xenoamicins\_biosynthetic\_gene\_cluster | 31 | 46.8914646997 | 166 | 1.9e-40 |
| CAE53351.1 | non-ribosomal\_peptide\_synthetase | BGC0000440 | Teicoplanin\_biosynthetic\_gene\_cluster | 27 | 67.1232876712 | 166 | 1.9e-40 |
| BAH22762.1 | nonribosomal\_peptide\_synthetase | BGC0001018 | Micropeptin\_biosynthetic\_gene\_cluster | 33 | 45.8377239199 | 166 | 1.9e-40 |
| AHY86403.1 | non\_ribosomal\_peptide\_synthetase | BGC0000329 | Corpeptin\_biosynthetic\_gene\_cluster | 26 | 90.727081138 | 165 | 2.5e-40 |
| AAC32048.1 | FxbC | BGC0000351 | Exochelin\_biosynthetic\_gene\_cluster | 27 | 69.0200210748 | 165 | 2.5e-40 |
| ABH06368.2 | MassB | BGC0000389 | Massetolide\_A\_biosynthetic\_gene\_cluster | 31 | 43.9409905163 | 164 | 5.6e-40 |
| AHZ34241.1 | CipD | BGC0001389 | Cichopeptin\_biosynthetic\_gene\_cluster | 32 | 44.5732349842 | 164 | 5.6e-40 |
| ALV86866.1 | Tlo20 | BGC0001406 | Telomycin\_biosynthetic\_gene\_cluster | 32 | 44.4678609062 | 164 | 7.3e-40 |
| CAM56770.1 |  | BGC0000354 | Friulimicin\_biosynthetic\_gene\_cluster | 31 | 44.1517386723 | 163 | 9.5e-40 |
| ALD82525.1 | non-ribosomal\_peptide\_synthase | BGC0001212 | Nannocystin\_biosynthetic\_gene\_cluster | 31 | 49.7365648051 | 163 | 1.2e-39 |
| AAF08797.1 | MycC | BGC0001103 | Mycosubtilin\_biosynthetic\_gene\_cluster | 31 | 45.8377239199 | 162 | 2.1e-39 |
| AAO56329.1 | non-ribosomal\_peptide\_synthetase\_SyfB | BGC0000435 | Syringafactin\_biosynthetic\_gene\_cluster | 31 | 45.2054794521 | 162 | 2.8e-39 |
| KPN90369.1 | NunE | BGC0001416 | Nunapeptin\_/\_nunamycin\_biosynthetic\_gene\_cluster | 29 | 46.9968387777 | 162 | 2.8e-39 |
| EFL06867.1 | predicted\_protein | BGC0000300 | Amychelin\_biosynthetic\_gene\_cluster | 32 | 47.2075869336 | 162 | 2.8e-39 |
| EAL92291.2 | nonribosomal\_peptide\_synthase\_GliP-like,\_putative | BGC0000372 | Hexadehydro-astechrome\_(HAS)\_biosynthetic\_gene\_cluster | 28 | 53.8461538462 | 161 | 3.6e-39 |
| AKA59447.1 | non-ribosomal\_peptide\_synthetase | BGC0001203 | Clarexpoxcin\_biosynthetic\_gene\_cluster | 33 | 45.7323498419 | 161 | 4.7e-39 |
| AGS06887.1 | mixed\_type\_I\_polyketide\_synthase/non-ribosomal\_peptide\_synthetase,\_PedF-like\_protein | BGC0001092\_c2 | Diaphorin\_biosynthetic\_gene\_cluster | 24 | 67.7555321391 | 161 | 4.7e-39 |
| CAJ18237.2 | non-ribosomal\_peptide\_synthetase\_B | BGC0000354 | Friulimicin\_biosynthetic\_gene\_cluster | 32 | 45.626975764 | 161 | 4.7e-39 |
| AAF00962.1 | McyC | BGC0001017 | Microcystin\_biosynthetic\_gene\_cluster | 30 | 44.9947312961 | 161 | 4.7e-39 |
| CDG17982.1 | Non-ribosomal\_peptide\_synthetase | BGC0000464 | Xenoamicins\_biosynthetic\_gene\_cluster | 32 | 45.9430979979 | 161 | 4.7e-39 |
| AFK57219.1 | DidH | BGC0000985 | Didemnin\_biosynthetic\_gene\_cluster | 29 | 68.5985247629 | 161 | 6.1e-39 |
| ADY76664.1 | non-ribosomal\_peptide\_synthetase | BGC0000950 | Napsamycin\_biosynthetic\_gene\_cluster | 32 | 44.5732349842 | 161 | 6.1e-39 |
| AEW31019.1 | plipastatin\_synthetase | BGC0000407 | Plipastatin\_biosynthetic\_gene\_cluster | 32 | 45.416227608 | 161 | 6.1e-39 |
| CAY48789.1 | putative\_non-ribosomal\_peptide\_synthetase | BGC0001312 | Viscosin\_biosynthetic\_gene\_cluster | 31 | 44.9947312961 | 161 | 6.1e-39 |
| AEI58867.1 | peptide\_synthetase | BGC0000455 | Vancomycin\_biosynthetic\_gene\_cluster | 32 | 44.7839831401 | 161 | 6.1e-39 |
| ADG27358.1 | peptide\_synthetase | BGC0000296 | Actinomycin\_biosynthetic\_gene\_cluster | 31 | 44.6786090622 | 160 | 1e-38 |
| AAT01807.1 | non-ribosomal\_peptide\_synthetase | BGC0000365 | Glycopeptidolipid\_biosynthetic\_gene\_cluster | 33 | 44.6786090622 | 160 | 1.4e-38 |
| AAC68816.1 | FK506\_peptide\_synthetase | BGC0000353 | FK506\_biosynthetic\_gene\_cluster | 27 | 66.2802950474 | 160 | 1.4e-38 |
| AEC14349.1 | nonribosomal\_peptide\_synthetase | BGC0000377 | Koranimine\_biosynthetic\_gene\_cluster | 31 | 42.9926238145 | 159 | 1.8e-38 |
| EAU36744.1 | predicted\_protein | BGC0000292 | Acetylaranotin\_biosynthetic\_gene\_cluster | 24 | 87.6712328767 | 159 | 1.8e-38 |
| ABD14712.1 | CesB | BGC0000320 | Cereulide\_biosynthetic\_gene\_cluster | 28 | 66.3856691254 | 159 | 1.8e-38 |
| AEP18655.1 | WAPS2 | BGC0000461 | WAP-8294A2\_(lotilibcin)\_biosynthetic\_gene\_cluster | 32 | 44.5732349842 | 159 | 1.8e-38 |
| CAA11796.1 | PCZA363.5 | BGC0000322 | Chloroeremomycin\_biosynthetic\_gene\_cluster | 31 | 45.416227608 | 159 | 2.3e-38 |
| ABL74940.1 | TlmVI | BGC0001048 | Tallysomycin\_biosynthetic\_gene\_cluster | 32 | 43.9409905163 | 159 | 2.3e-38 |
| ACM68690.1 | AerG1 | BGC0000298 | Aeruginosin\_biosynthetic\_gene\_cluster | 31 | 44.2571127503 | 159 | 2.3e-38 |
| AHB82072.1 | non\_ribosomal\_peptide\_synthetase/polyketide\_synthase | BGC0001231 | Microsclerodermins\_biosynthetic\_gene\_cluster | 32 | 44.6786090622 | 159 | 2.3e-38 |
| CAN89664.1 | putative\_amino\_acid\_adenylating\_enzyme | BGC0001070 | Kirromycin\_biosynthetic\_gene\_cluster | 32 | 45.7323498419 | 158 | 3e-38 |
| ABV79986.1 | ApnB | BGC0000301 | Anabaenopeptin\_biosynthetic\_gene\_cluster | 30 | 44.3624868282 | 158 | 3e-38 |
| WP\_028678148.1 | non-ribosomal\_peptide\_synthetase | BGC0001228 | Retimycin\_biosynthetic\_gene\_cluster | 33 | 45.1001053741 | 158 | 3e-38 |
| AGZ15460.1 | putative\_non-ribosomal\_peptide\_synthetase | BGC0001036 | Polyoxypeptin\_biosynthetic\_gene\_cluster | 30 | 42.5711275026 | 158 | 3e-38 |
| AAZ23078.1 | peptide\_synthetase | BGC0000291 | A54145\_biosynthetic\_gene\_cluster | 31 | 45.626975764 | 158 | 3e-38 |
| AHB82059.1 | non\_ribosomal\_peptide\_synthetase/polyketide\_synthase | BGC0001019 | Microsclerodermins\_biosynthetic\_gene\_cluster | 31 | 46.2592202318 | 157 | 6.8e-38 |
| CCP45167.1 | Peptide\_synthetase\_MbtF\_(peptide\_synthase) | BGC0001021 | Mycobactin\_biosynthetic\_gene\_cluster | 32 | 45.31085353 | 157 | 6.8e-38 |
| ADG27359.1 | peptide\_synthetase | BGC0000296 | Actinomycin\_biosynthetic\_gene\_cluster | 31 | 44.8893572181 | 157 | 6.8e-38 |
| ADI59531.1 | CorI | BGC0001091 | Corallopyronin\_biosynthetic\_gene\_cluster | 32 | 42.4657534247 | 157 | 8.9e-38 |
| ABC36785.1 | peptide\_synthetase,\_putative | BGC0000964 | Burkholdac\_biosynthetic\_gene\_cluster | 31 | 45.9430979979 | 156 | 1.5e-37 |
| CAL80821.1 | sylD-like\_NRPS/PKS | BGC0000997 | Glidobactin\_biosynthetic\_gene\_cluster | 34 | 42.0442571128 | 156 | 1.5e-37 |
| CDF96614.1 | Long-chain-fatty-acid--CoA\_ligase | BGC0001149 | Brabantamide\_biosynthetic\_gene\_cluster | 26 | 69.3361433087 | 156 | 2e-37 |
| AFK57212.1 | DidA | BGC0000985 | Didemnin\_biosynthetic\_gene\_cluster | 29 | 68.4931506849 | 156 | 2e-37 |
| ADZ45324.1 | non-ribosomal\_peptide\_synthetase | BGC0001020 | Muraymycin\_biosynthetic\_gene\_cluster | 29 | 63.751317176 | 155 | 3.4e-37 |
| ABR67750.1 | CmnG | BGC0000316 | Capreomycin\_biosynthetic\_gene\_cluster | 27 | 69.5468914647 | 155 | 3.4e-37 |
| CCE28989.1 | non-ribosomal\_peptide\_synthetase | BGC0001365 | Clapurines\_biosynthetic\_gene\_cluster | 31 | 43.6248682824 | 155 | 4.4e-37 |
| BAL15726.1 | NRPS | BGC0000432 | Streptothricin\_biosynthetic\_gene\_cluster | 31 | 47.5237091675 | 154 | 5.7e-37 |
| ABV56582.1 | KtzB | BGC0000378 | Kutznerides\_biosynthetic\_gene\_cluster | 28 | 44.4678609062 | 154 | 7.5e-37 |
| AGS77310.1 | NRPS\_module\_7 | BGC0001178 | UK-68,597\_biosynthetic\_gene\_cluster | 30 | 45.626975764 | 154 | 7.5e-37 |
| AAT01806.1 | non-ribosomal\_peptide\_synthetase | BGC0000365 | Glycopeptidolipid\_biosynthetic\_gene\_cluster | 32 | 42.5711275026 | 154 | 7.5e-37 |
| CAJ34382.1 | NRPS\_protein | BGC0000445 | Thiocoraline\_biosynthetic\_gene\_cluster | 29 | 45.2054794521 | 153 | 9.8e-37 |
| AJF34464.1 | Txo2 | BGC0001207 | Teixobactin\_biosynthetic\_gene\_cluster | 30 | 45.8377239199 | 153 | 1.3e-36 |
| AEO14743.1 | NdaA | BGC0000396 | Nodularin\_biosynthetic\_gene\_cluster | 29 | 51.2118018967 | 153 | 1.3e-36 |
| AAZ03551.1 | McnB | BGC0000332 | Cyanopeptolin\_biosynthetic\_gene\_cluster | 30 | 47.9452054795 | 153 | 1.7e-36 |
| AHB82071.1 | non\_ribosomal\_peptide\_synthetase | BGC0001231 | Microsclerodermins\_biosynthetic\_gene\_cluster | 30 | 45.626975764 | 153 | 1.7e-36 |
| CAN89656.1 | putative\_non-ribosomal\_peptide\_synthetase | BGC0001070 | Kirromycin\_biosynthetic\_gene\_cluster | 31 | 45.521601686 | 152 | 2.2e-36 |
| CAJ34375.1 | NRPS | BGC0000445 | Thiocoraline\_biosynthetic\_gene\_cluster | 29 | 45.31085353 | 152 | 2.9e-36 |
| CAQ18831.1 | nonribosomal\_polypetide\_synthetase | BGC0000954 | Ajudazol\_biosynthetic\_gene\_cluster | 30 | 45.31085353 | 151 | 3.7e-36 |
| AAG02364.1 | peptide\_synthetase\_NRPS2-1 | BGC0000963 | Bleomycin\_biosynthetic\_gene\_cluster | 30 | 44.6786090622 | 151 | 6.4e-36 |
| AAZ55898.1 | amino\_acid\_adenylation | BGC0000359 | Fuscachelin\_biosynthetic\_gene\_cluster | 32 | 46.7860906217 | 150 | 8.3e-36 |
| ABG00046.1 | non-ribosomal\_peptide\_synthetase | BGC0000387 | Mangotoxin\_biosynthetic\_gene\_cluster | 30 | 43.2033719705 | 150 | 8.3e-36 |
| CAE53353.1 | non-ribosomal\_peptide\_synthetase | BGC0000440 | Teicoplanin\_biosynthetic\_gene\_cluster | 31 | 44.6786090622 | 150 | 8.3e-36 |
| AHD05616.1 | putative\_non-ribosomal\_peptide\_ligase\_domain\_protein | BGC0001033 | Paenilamicin\_biosynthetic\_gene\_cluster | 26 | 72.813487882 | 150 | 1.1e-35 |
| CAD91211.1 | putative\_non-ribosomal\_peptide\_synthetase,\_module\_7 | BGC0000289 | A40926\_biosynthetic\_gene\_cluster | 30 | 43.7302423604 | 150 | 1.1e-35 |
| AMK48226.1 | nonribosomal\_peptide\_synthetase | BGC0001351 | Cahuitamycins\_biosynthetic\_gene\_cluster | 33 | 44.4678609062 | 149 | 1.4e-35 |
| AGZ15476.1 | putative\_non\_ribosomal\_peptide\_synthetase | BGC0001036 | Polyoxypeptin\_biosynthetic\_gene\_cluster | 27 | 65.5426765016 | 149 | 1.8e-35 |
| CAG15012.1 | peptide\_synthetase,\_module\_7 | BGC0000441 | Teicoplanin\_biosynthetic\_gene\_cluster | 31 | 43.8356164384 | 148 | 3.2e-35 |
| CAC48362.1 | peptide\_synthetase | BGC0000311 | Balhimycin\_biosynthetic\_gene\_cluster | 30 | 43.9409905163 | 148 | 4.1e-35 |
| AID65224.1 | nonribosomal\_peptide\_synthetase | BGC0000335 | Cystomanamides\_biosynthetic\_gene\_cluster | 31 | 44.3624868282 | 146 | 1.2e-34 |
| CAF05648.1 | TubC\_protein | BGC0001053 | Tubulysin\_biosynthetic\_gene\_cluster | 30 | 43.9409905163 | 146 | 1.2e-34 |
| CAJ14037.1 | peptide\_synthetase\_PhsB | BGC0000406 | Phosphinothricin\_biosynthetic\_gene\_cluster | 31 | 44.3624868282 | 146 | 1.2e-34 |
| AAK81826.1 | peptide\_synthetase | BGC0000326 | Complestatin\_biosynthetic\_gene\_cluster | 29 | 45.31085353 | 146 | 1.6e-34 |
| CBW45740.1 | hybrid\_NRPS/PKS\_(PKSIV) | BGC0000952\_c3 | Pristinamycin\_biosynthetic\_gene\_cluster | 26 | 69.1253951528 | 146 | 1.6e-34 |
| AFV27434.1 | indigoidine\_synthase | BGC0000375 | Indigoidine\_biosynthetic\_gene\_cluster | 28 | 50.3688092729 | 146 | 2e-34 |
| ABP55216.1 | AMP-dependent\_synthetase\_and\_ligase | BGC0000142 | Salinilactam\_biosynthetic\_gene\_cluster | 28 | 56.3751317176 | 145 | 2.7e-34 |
| AEA30272.1 | peptide\_synthetase | BGC0000429 | Skyllamycin\_biosynthetic\_gene\_cluster | 28 | 48.8935721812 | 145 | 2.7e-34 |
| BAE56606.1 |  | BGC0001123 | Aspirochlorine\_biosynthetic\_gene\_cluster | 30 | 44.3624868282 | 144 | 4.6e-34 |
| AHZ20784.1 | non-ribosomal\_peptide\_synthase | BGC0000369 | Hassallidins\_biosynthetic\_gene\_cluster | 29 | 45.9430979979 | 144 | 5.9e-34 |
| CAH55654.1 | putative\_L-prolyl-AMP\_ligase | BGC0000259 | Prodigiosin\_biosynthetic\_gene\_cluster | 27 | 56.0590094837 | 144 | 7.8e-34 |
| CAG29031.1 | nonribosomal\_peptide\_synthetase\_(modules\_1\_and\_2) | BGC0001023 | Myxochromide\_biosynthetic\_gene\_cluster | 29 | 50.2634351949 | 144 | 7.8e-34 |
| CAD70195.1 | non-ribosomal\_peptide\_synthetase | BGC0001047 | Syringolin\_A\_biosynthetic\_gene\_cluster | 31 | 41.7281348788 | 143 | 1e-33 |
| AAQ90177.1 | putative\_acyl-CoA\_synthetase | BGC0000128 | Pyoluteorin\_biosynthetic\_gene\_cluster | 30 | 44.9947312961 | 143 | 1e-33 |
| KFH48607.1 | N-like\_protein | BGC0000317 | Cephalosporin\_biosynthetic\_gene\_cluster | 29 | 46.7860906217 | 143 | 1.3e-33 |
| AAC83656.1 | dihydroaeruginoic\_acid\_synthetase | BGC0000412 | Pyochelin\_biosynthetic\_gene\_cluster | 30 | 44.6786090622 | 143 | 1.7e-33 |
| ABR67758.1 | CmnO | BGC0000316 | Capreomycin\_biosynthetic\_gene\_cluster | 30 | 44.9947312961 | 142 | 2.3e-33 |
| BAO66530.1 | nonribosomal\_peptide\_synthase | BGC0000042 | Cremimycin\_biosynthetic\_gene\_cluster | 30 | 45.7323498419 | 142 | 3e-33 |
| BAF50727.1 | hybrid\_polyketide\_synthase-non\_ribosomal\_peptide\_synthetase | BGC0001116 | Virginiamycin\_biosynthetic\_gene\_cluster | 30 | 43.7302423604 | 141 | 3.9e-33 |
| CBD77749.1 | non-ribosomal\_peptide\_synthetase | BGC0000974 | Crocacin\_biosynthetic\_gene\_cluster | 31 | 43.5194942044 | 141 | 5e-33 |
| ABW71852.1 | nonribosomal\_peptide\_synthetase | BGC0000303 | Anthramycin\_biosynthetic\_gene\_cluster | 26 | 56.6912539515 | 140 | 8.6e-33 |
| CAE14173.1 |  | BGC0000383 | Luminmycin\_biosynthetic\_gene\_cluster | 31 | 41.8335089568 | 140 | 1.1e-32 |
| AAF63833.1 | PstD | BGC0000362 | Glycopeptidolipid\_biosynthetic\_gene\_cluster | 32 | 43.7302423604 | 139 | 1.5e-32 |
| AAG05690.1 | AmbE | BGC0000287 | 2-amino-4-methoxy-trans-3-butenoic\_acid\_biosynthetic\_gene\_cluster | 29 | 47.3129610116 | 139 | 1.9e-32 |
| ALA09366.1 | AMP-dependent\_synthetase\_and\_ligase | BGC0001303 | Lobosamide\_biosynthetic\_gene\_cluster | 32 | 45.31085353 | 139 | 2.5e-32 |
| AHD05621.1 | non-ribosomal\_peptide\_ligase\_domain\_protein | BGC0001033 | Paenilamicin\_biosynthetic\_gene\_cluster | 27 | 51.8440463646 | 138 | 5.6e-32 |
| ADQ55475.1 | non-ribosomal\_peptide\_synthase\_module | BGC0000350 | ET-743\_biosynthetic\_gene\_cluster | 26 | 45.521601686 | 138 | 5.6e-32 |
| ADQ55476.1 | non-ribosomal\_peptide\_synthase\_dimodule | BGC0000350 | ET-743\_biosynthetic\_gene\_cluster | 23 | 69.3361433087 | 138 | 5.6e-32 |
| CAE52339.1 | Polyketide\_non-ribosomal\_peptide\_synthase | BGC0001088 | Albicidin\_biosynthetic\_gene\_cluster | 28 | 46.8914646997 | 137 | 9.5e-32 |
| CBW45640.1 | PII\_peptide\_synthetase | BGC0000952 | Pristinamycin\_biosynthetic\_gene\_cluster | 30 | 46.4699683878 | 136 | 1.2e-31 |
| CDG12864.1 | non-ribosomal\_peptide\_synthetase | BGC0001415 | Althiomycin\_biosynthetic\_gene\_cluster | 27 | 46.1538461538 | 135 | 3.6e-31 |
| AAN85506.1 | nonribosomal\_peptide\_synthetase | BGC0001101 | Leinamycin\_biosynthetic\_gene\_cluster | 30 | 45.521601686 | 134 | 4.7e-31 |
| ADL64235.1 | aureusimine\_non-ribosomal\_peptide\_synthetase | BGC0000308 | Aureusimine\_biosynthetic\_gene\_cluster | 30 | 44.3624868282 | 134 | 6.2e-31 |
| BAR73011.1 | putative\_ATP-dependent\_aminoacyl-ACP\_synthetase | BGC0001194 | Hitachimycin\_biosynthetic\_gene\_cluster | 30 | 43.4141201264 | 134 | 6.2e-31 |
| EAW09117.1 | hybrid\_NRPS/PKS\_enzyme,\_putative | BGC0000983 | Cytochalasin\_biosynthetic\_gene\_cluster | 27 | 66.8071654373 | 134 | 8e-31 |
| AGI61651.1 | long\_chain\_fatty\_acid\_CoA\_ligase | BGC0000095 | Micromonolactam\_biosynthetic\_gene\_cluster | 27 | 56.5858798736 | 133 | 1e-30 |
| ACO94492.1 | NRPS\_adenylation\_domain\_protein | BGC0000097 | ML-449\_biosynthetic\_gene\_cluster | 30 | 42.4657534247 | 131 | 4e-30 |
| ADN43685.1 | DmbS | BGC0001136 | Desmethylbassianin\_biosynthetic\_gene\_cluster | 29 | 46.2592202318 | 129 | 1.5e-29 |
| ctg1\_orf10 |  | BGC0000321 | Chaetocin\_biosynthetic\_gene\_cluster | 24 | 93.8883034773 | 129 | 2e-29 |
| CAF32362.1 | putative\_non-ribosomal\_peptide\_synthetase | BGC0000712 | Paromomycin\_biosynthetic\_gene\_cluster | 29 | 46.0484720759 | 128 | 3.4e-29 |
| CAD29794.1 | peptide\_synthetase | BGC0001015 | Microcystin\_biosynthetic\_gene\_cluster | 28 | 45.9430979979 | 128 | 5.8e-29 |
| EWS95124.1 | hypothetical\_protein | BGC0000306 | Arylomycin\_biosynthetic\_gene\_cluster | 34 | 28.8724973656 | 126 | 1.3e-28 |
| BAP34718.1 | AMP-dependent\_synthetase\_and\_ligase | BGC0000078 | Incednine\_biosynthetic\_gene\_cluster | 30 | 45.8377239199 | 126 | 1.7e-28 |
| AEC14347.1 | nonribosomal\_peptide\_synthetase | BGC0000377 | Koranimine\_biosynthetic\_gene\_cluster | 27 | 44.6786090622 | 125 | 3.7e-28 |
| ACO94464.1 | NRPS\_adenylation\_domain\_protein | BGC0000029 | BE-14106\_biosynthetic\_gene\_cluster | 30 | 42.3603793467 | 125 | 3.7e-28 |
| CAH55637.1 | putative\_L-prolyl-AMP\_ligase | BGC0000258 | Prodigiosin\_biosynthetic\_gene\_cluster | 28 | 56.6912539515 | 123 | 1.4e-27 |
| BAD08370.1 | non-ribosomal\_peptide\_synthetase | BGC0000167 | Vicenistatin\_biosynthetic\_gene\_cluster | 28 | 44.0463645943 | 123 | 1.4e-27 |
| AKD43757.1 | HerL | BGC0001349 | Heronamide\_biosynthetic\_gene\_cluster | 30 | 42.6765015806 | 123 | 1.9e-27 |
| ADB02873.1 | AzicV | BGC0000202 | Azicemicin\_biosynthetic\_gene\_cluster | 25 | 54.478398314 | 120 | 1.2e-26 |
| CAA60461.1 | pipecolate\_incorporating\_enzyme | BGC0001040 | Rapamycin\_biosynthetic\_gene\_cluster | 27 | 45.1001053741 | 120 | 1.2e-26 |
| AKC54422.1 | fumosorinone\_biosynthesis\_polyketide\_synthase | BGC0001218 | Fumosorinone\_biosynthetic\_gene\_cluster | 26 | 66.4910432034 | 119 | 2e-26 |
| XP\_002384042.1 | NRPS-like\_enzyme,\_putative | BGC0001234 | 4,4'-piperazine-2,5-diyldimethyl-bis-phenol\_biosynthetic\_gene | 27 | 48.4720758693 | 119 | 2e-26 |
| AHB38515.1 | EpnG | BGC0000345 | Eponemycin\_biosynthetic\_gene\_cluster | 27 | 44.6786090622 | 119 | 2.7e-26 |
| XP\_002384859.1 | NRPS-like\_enzyme,\_putative | BGC0001234\_c2 | 4,4'-piperazine-2,5-diyldimethyl-bis-phenol\_biosynthetic\_gene | 26 | 46.4699683878 | 118 | 6e-26 |
| CBJ89771.1 | Non-ribosomal\_peptide\_synthase\_involved\_in\_Xenocoumacin\_synthesis | BGC0001054 | Xenocoumacin\_biosynthetic\_gene\_cluster | 25 | 45.8377239199 | 114 | 8.6e-25 |
| AAS98783.1 | JamL | BGC0001001 | Jamaicamide\_biosynthetic\_gene\_cluster | 30 | 31.5068493151 | 113 | 1.5e-24 |
| CAJ34370.1 | NRPS\_protein | BGC0000445 | Thiocoraline\_biosynthetic\_gene\_cluster | 28 | 41.5173867229 | 112 | 3.3e-24 |
| CCA54209.1 | non-ribosomal\_peptide\_synthetase | BGC0000893 | Chloramphenicol\_biosynthetic\_gene\_cluster | 30 | 44.3624868282 | 111 | 4.3e-24 |
| AAQ93484.1 | CmaA | BGC0000328 | Coronatine\_biosynthetic\_gene\_cluster | 24 | 61.9599578504 | 111 | 5.6e-24 |
| EJK79843.1 | amino\_acid\_adenylation\_enzyme/thioester\_reductase\_family\_protein | BGC0000436 | Syringolin\_A\_biosynthetic\_gene\_cluster | 28 | 46.4699683878 | 109 | 2.1e-23 |
| ABY83146.1 | Azi7 | BGC0000960 | Azinomycin\_B\_biosynthetic\_gene\_cluster | 28 | 45.521601686 | 108 | 4.7e-23 |
| BAO66533.1 | nonribosomal\_peptide\_synthase | BGC0000042 | Cremimycin\_biosynthetic\_gene\_cluster | 27 | 54.373024236 | 105 | 4e-22 |
| XP\_001220460.1 | hypothetical\_protein | BGC0001182 | Chaetoglobosins\_biosynthetic\_gene\_cluster | 24 | 70.0737618546 | 103 | 1.5e-21 |
| CCB53264.1 | non-ribosomal\_peptide\_synthetase | BGC0001393 | Lugdunin\_biosynthetic\_gene\_cluster | 27 | 28.9778714436 | 101 | 5.8e-21 |
| CBW45681.1 | putative\_amino\_acid\_adenyltransferase | BGC0000952\_c2 | Pristinamycin\_biosynthetic\_gene\_cluster | 32 | 30.3477344573 | 99 | 2.9e-20 |
| BAR73008.1 | putative\_ATP-dependent\_b-aminoacyl-ACP\_synthetase | BGC0001194 | Hitachimycin\_biosynthetic\_gene\_cluster | 27 | 57.7449947313 | 94 | 7.1e-19 |
| AFP87519.1 | proline\_adenyltransferase | BGC0001159 | Marinopyrrole\_biosynthetic\_gene\_cluster | 29 | 30.2423603793 | 94 | 9.2e-19 |
| EFY94493.1 | nonribosomal\_peptide\_synthase\_GliP-like,\_putative | BGC0000337 | Destruxins\_biosynthetic\_gene\_cluster | 24 | 57.7449947313 | 92 | 3.5e-18 |
| ACO94461.1 | putative\_AMP-dependent\_acyl-CoA\_synthetase/ligase | BGC0000029 | BE-14106\_biosynthetic\_gene\_cluster | 25 | 55.4267650158 | 90 | 1e-17 |
| AKD43754.1 | HerJ | BGC0001349 | Heronamide\_biosynthetic\_gene\_cluster | 26 | 43.3087460485 | 87 | 6.6e-17 |
| BAP34707.1 | AMP-dependent\_synthetase\_and\_ligase | BGC0000078 | Incednine\_biosynthetic\_gene\_cluster | 26 | 47.1022128556 | 87 | 8.6e-17 |
| ACO94489.1 | putative\_AMP-dependent\_acyl-CoA\_synthetase/ligase | BGC0000097 | ML-449\_biosynthetic\_gene\_cluster | 26 | 43.2033719705 | 87 | 8.6e-17 |
| AMK48234.1 | salicylate-AMP\_ligase | BGC0001351 | Cahuitamycins\_biosynthetic\_gene\_cluster | 24 | 45.8377239199 | 82 | 3.6e-15 |
| CAJ34366.1 | putative\_3-hydroxy-quinaldate-AMP-Ligase | BGC0000445 | Thiocoraline\_biosynthetic\_gene\_cluster | 23 | 57.5342465753 | 81 | 4.7e-15 |
| AAO07759.1 | 2,3-dihydroxybenzoate-AMP\_ligase | BGC0000460 | Vulnibactin\_biosynthetic\_gene\_cluster | 25 | 28.7671232877 | 81 | 6.2e-15 |
| CAC17498.1 | putative\_AMP-binding\_ligase | BGC0000324 | Coelibactin\_biosynthetic\_gene\_cluster | 28 | 29.9262381454 | 80 | 1.1e-14 |
| ABP55217.1 | AMP-dependent\_synthetase\_and\_ligase | BGC0000142 | Salinilactam\_biosynthetic\_gene\_cluster | 24 | 47.1022128556 | 77 | 6.8e-14 |
| CAN89663.1 | putative\_non-ribosomal\_peptide\_synthetase | BGC0001070 | Kirromycin\_biosynthetic\_gene\_cluster | 26 | 32.1390937829 | 76 | 1.5e-13 |
| AGI61652.1 | long\_chain\_fatty\_acid\_CoA\_ligase | BGC0000095\_c2 | Micromonolactam\_biosynthetic\_gene\_cluster | 25 | 46.4699683878 | 76 | 1.5e-13 |
| EFL06871.1 | 2,3-dihydroxybenzoate-AMP\_ligase | BGC0000300 | Amychelin\_biosynthetic\_gene\_cluster | 24 | 44.8893572181 | 76 | 2.6e-13 |
| AAO39105.1 | AdmK | BGC0000956 | Andrimid\_biosynthetic\_gene\_cluster | 26 | 42.5711275026 | 76 | 2.6e-13 |
| ACU36660.1 | AMP-dependent\_synthetase\_and\_ligase | BGC0000392 | Mirubactin\_biosynthetic\_gene\_cluster | 24 | 46.6807165437 | 75 | 3.4e-13 |
| ALA09365.1 | AMP-dependent\_synthetase\_and\_ligase | BGC0001303 | Lobosamide\_biosynthetic\_gene\_cluster | 24 | 46.5753424658 | 74 | 9.9e-13 |
| CAA79245.2 | enniatin\_synthetase | BGC0000342 | Enniatin\_biosynthetic\_gene\_cluster | 24 | 45.8377239199 | 73 | 1.3e-12 |
| AAL01056.1 | putative\_acyl-CoA\_ligase | BGC0000865 | Eicoseicosapentaenoic\_acid\_biosynthetic\_gene\_cluster | 23 | 29.9262381454 | 52 | 4e-06 |
